# Positive Influence of Stroke Health Manager on Risk Factors Control and Medication Adherence After Ischemic Stroke

**DOI:** 10.3389/fneur.2020.00168

**Published:** 2020-03-11

**Authors:** Xiuli Yan, Zhuo Liu, Zhen-Ni Guo, Ye Sun, Hang Jin, Xin Sun, Huijie Sun, Yi Yang

**Affiliations:** ^1^Department of Neurology, The First Hospital of Jilin University, Changchun, China; ^2^Cadre Ward, The First Hospital of Jilin University, Changchun, China

**Keywords:** stroke health manager, ischemic stroke, secondary prevention, compliance, risk factors

## Abstract

**Background:** From 2017, the Stroke Health Manager Training Project was carried out by the Chinese Government to strengthen health management and follow-up intervention after ischemic stroke. The aim of this study was to investigate whether after the intervention of the stroke health manager, the control of blood pressure, low-density lipoprotein cholesterol (LDL-C), glucose level, and the use of secondary prevention medications improved 3 months after discharge from our center following ischemic stroke.

**Methods:** The study used a history-controlled approach. Patients who received stroke health manager intervention from May 1, 2018, to March 31, 2019, were considered as the intervention group; those from May 1, 2017, to April 30, 2018, were enrolled as the control group. Stroke health manager intervention included health education, discharge advice, online WeChat public group follow-up, and clinical consultation.

**Results:** In total, 642 patients with ischemic stroke were enrolled in this study (277 in the control group, 365 in the intervention group). At 3 months, the blood pressure, LDL-C and glucose control in the intervention group were better than in the control group (all *P* < 0.05). At the same time, the overall persistence for secondary prevention medications at 3 months after discharge increased from 201/277 (72.56%) to 303/365 (83.01%, *P* = 0.001). The persistence for patients taking antiplatelet, hypoglycemic and statins were significantly higher in the intervention group (*P* < 0.05).

**Conclusions:** Stroke health manager intervention improved the control of blood pressure, LDL-C, glucose levels and the persistence for secondary prevention medications 3 months after discharge.

## Introduction

Stroke is the leading cause of death in China (1). China's National Stroke Registry investigated patients who had previously experienced acute ischemic stroke and found that the 1-year recurrence rate of stroke was 17.7% (2). Stroke recurrence leads to aggravation of existing neurological dysfunction and a significant increase in mortality; indeed, recurrence has always been one of the key issues to be solved in the prevention and treatment of this disease.

For stroke patients, strengthening health management to control risk factors and focusing on compliance with secondary prevention are critical (3–5). However, in China, the compliance of stroke patients with risk factor control and secondary prevention is unsatisfactory (6–8). In September 2017, in order to strengthen health management and secondary prevention, the Stroke Prevention and Treatment Engineering Committee of the National Health Commission of the People's Republic of China launched the Stroke Health Manager Training Project, with the aim of carrying out the training work for stroke health manager in the National Stroke Screening and Prevention Base and Senior Stroke Center Unit. Upon passing an examination, qualified individuals receive government-issued certification for stroke health management.

The present study aimed to verify the role of the stroke health manager in the prevention and the control of stroke via comparing the blood pressure, LDL-C and glucose control, as well as determining the persistence for secondary prevention medications in stroke patients after intervention by the stroke health manager.

## Methods

The study design was approved by the ethics committee of the First Hospital of Jilin University. All participants gave written informed consent. Data that support the findings of this study are available from the corresponding author on reasonable request.

## Study Design and Participants

This was a history-controlled study with two groups. Based on the electronic medical records, patients were consecutively recruited from May 1, 2017, to March 31, 2019 by a senior nurse, if they met the following criteria: (1) age ≥ 18 years; (2) hospitalized with a primary diagnosis of ischemic stroke, with the diagnosis being made according to the Guidelines for the Early Management of Patients with Acute Ischemic Stroke, (9) and with CT or MRI confirmation; (3) finished the face-to-face follow-up 3 months after hospital discharge; (4) gave, or authorized a representative to give informed consent. After excluding patients with incomplete follow-up information, the patients were divided into a control group (May 1, 2017–April 30, 2018) and intervention group (May 1, 2018–March 31, 2019) according to the induction of the stroke health manager.

## Health Management

### Stroke Health Manager

From the government report (10): Stroke health manager is a brand-new high-end profession for which a senior nurse is recommended. Stroke health management is based on evidence-based medicine, using multidisciplinary and safe-drug-use knowledge and a variety of modern management methods, aiming at the health status and risk factors of individuals and groups, carrying out health education and reducing or delaying the occurrence of diseases, improving people's quality of life and prolonging healthy life in order to achieve a reduction in medical expenses and social medical costs, and serving as a bridge and coordination between scientific research and health management.

**Figure 1:**
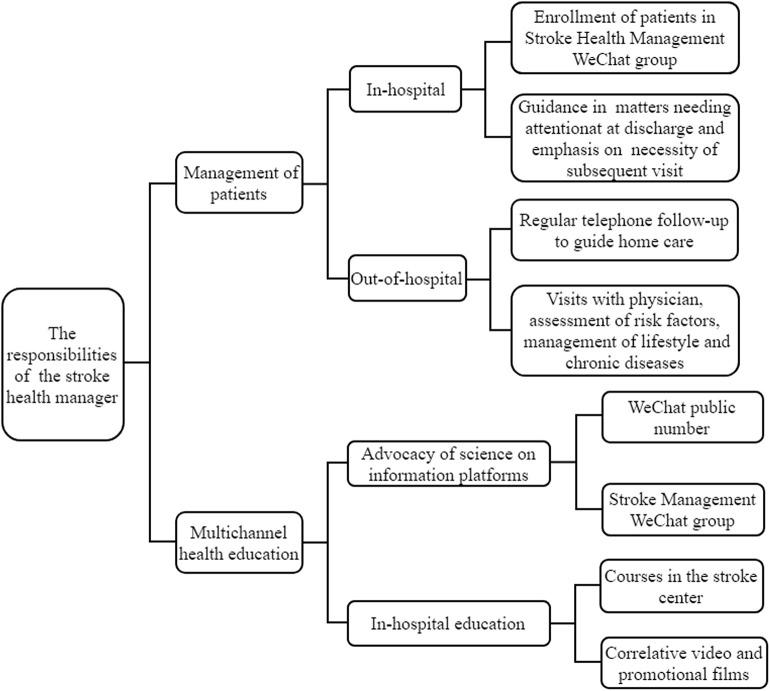
The responsibilities of the stroke health manager.

A senior nurse with rich clinical experience in our department was selected to participate in the Health China 2030 Stroke Health Manager Training Project. Certification was issued upon passing an examination after 3 weeks of multidisciplinary training. Based in the post-stroke follow-up and health-management clinic in our hospital, the stroke health manager provided all-round, one-stop, scientific health-management services, including health education, discharge advice, We Chat public group follow-up, and clinical consultation. The management of patients after discharge was carried out through WeChat public group, telephone follow-up, and face-to-face follow-up. Telephone follow-up is generally conducted 3, 6, 12 months after discharge for patients who have not been followed up in the follow-up clinic, which was conducted by trained neurologists and secretaries in our department, including self-care ability, drug use, etc. Multi-channel health education demonstrates the form of health education we use for both hospitalized and discharged patients (Figure 1).

### Health Education

Every workday afternoon, the stroke health manager used multimedia devices to provide half-hour courses in the stroke center for all hospitalized stroke patients and their family members. The contents were based on those proposed by American Heart Association/American Stroke Association Guidelines (5). This included management of stroke risk factors, prevention of deep vein thrombosis in the lower extremities, healthy dietary guidance, early exercise rehabilitation therapy, and health education on constipation. It was among the stroke health manager's responsibilities to ensure the quality and continuity of educational content so that patients could learn more about stroke prevention and care during hospitalization; as well as to make a correlative video and promotional films (e.g., ankle pump exercises), which were to be played on the display screen of the outpatient hall and treatment area.

### Discharge Advice

The stroke health manager issued a follow-up notice to patients who planned to be discharged from the hospital, informing them of relevant matters and filling in the date of the subsequent visit. The stroke health manager also provided a follow-up manual to the patients, which contained personal information, discharged medication, precautions, follow-up records, emphasized regular medication, marked the risk factors of the disease, and explained the control points in condensed (for example, patients with high blood pressure need to eat food with less oil and salt, keep a stable mood and do moderate exercise. For patients with hyperglycemia, a guide is provided for them to choose the appropriate food).

### Online WeChat Public Group

**Figure 2:**
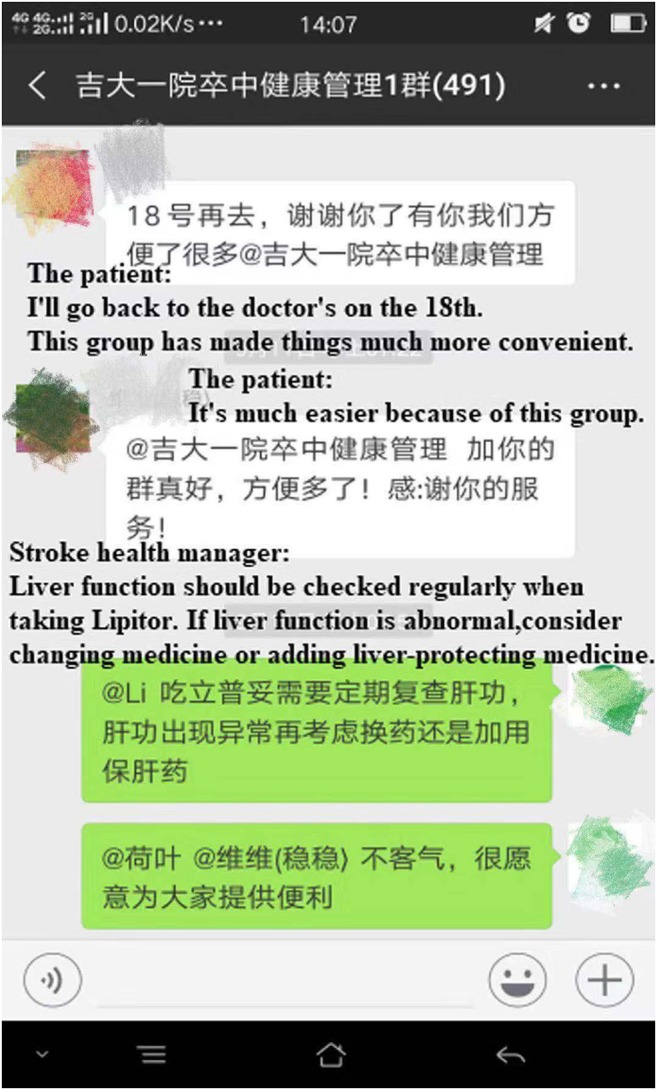
Chat records of stroke health management WeChat group (the permissions of the relevant personnel has been obtained).

In order to strengthen the continuing management of discharged patients, the stroke health manager established a WeChat public group of stroke patients, which sent daily stroke-related health knowledge, answered consultation related to stroke, smoothed the channel of reexamination, urged the patients to carry out the subsequent visits, assisted in risk factors management and medication compliance, and guided home care. At present, three WeChat groups have been established, with a total of more than 1,230 people, including patients in the intervention group and some patients who did not meet the study inclusion criteria, they could communicate with each other, as well as with stroke health manager (Figure 2). A WeChat public account for the Cerebrovascular Disease Center was also established. The stroke health manager also published two articles a month on stroke-related knowledge.

### Clinical Consultation

The Department of Neurology at the First Hospital of Jilin University established a post-stroke follow-up clinic in May 2017. The routine was to inform patients to come to the clinic at 3 months, 6 months, and 1 year after discharge. If a patient wishes to seek counseling beyond the prescribed follow-up time, they can contact the health manager through WeChat group to make an appointment for clinical consultation. The stroke health manager visited with a senior neurologist every weekday to provide an individualized non-drug control scheme, measured the blood pressure, blood glucose and other biochemical parameters, and evaluated patients' risk of recurrence. The patients were then given further management plans, including those to correct poor lifestyle choices and those for medication guidance.

The control group received standard medical care during hospitalization. The usual care only consisted of advice on healthy lifestyle choices carried out by primary nurse, but no structured education, WeChat group or clinical consultation was provided.

## Data Collection and Outcome Assessment

The physician in the clinic used a paper-based registry form for study-data collection. We used the results of a patient's examination the day before discharge recorded in the medical record information as the baseline information. Results measured in the follow-up clinic at 3 months after discharge were used as outcome indicators, which included sitting systolic blood pressure (SBP), diastolic blood pressure (DBP), blood lipids, modified Rankin score (mRS), and recurrence rate. Blood pressure was measured once in the seated position after 5 min of rest. An mRS score ≤ 2 at 3 months post-discharge was defined as a good functional outcome. Barthe index ≥ 60 was defined as basic self-care in daily life. Fasting blood glucose was not a routine examination for all patients, and only patients with a history of diabetes as listed in the follow-up manual were given a blood glucose examination during follow-up. Blood pressure and blood glucose levels were compared only between hypertensive and diabetic patients. Six months after discharge, the patient was followed up over telephone by the health manager to see if there was any recurrence or readmission. All the data was entered into a specialized database.

Several evidence-based secondary preventive medications were investigated based on the guidelines (5, 11): antiplatelet agents (acetylsalicylic acid, clopidogrel), antihypertensive agents (beta-blockers, angiotensin converting enzyme inhibitors, angiotensin receptor blockers, calcium-channel blockers, and diuretics), statins, and hypoglycemic agents (insulin and oral diabetic agents). The discontinuation of medication by a subject was ascertained by comparing hospital discharge medications (copies of the discharge records) with the current medications used, obtained by self-report. Three-month persistence was defined as continuation of all secondary preventive medications prescribed at discharge (12). Patients were considered non-persistent if they were prescribed an individual medication at hospital discharge but failed to take that medication during the 3 months post-discharge. However, subjects were considered persistent if there was a switch of medication within the same class.

## Statistical Methods

The chi-square test was used to compare categorical variables, and the independent sample *t*-tests were used to compare continuous variables in accordance with the normal distribution, which expressed as the mean ± standard deviation (x ± s). The measurement data that do not conform to a normal distribution are described by median and quartile range, whereas the comparison for the two groups used Mann-Whitney U test. Missing data were not replaced. Univariate variables with a *P*-value <0.2 were retained in a multivariate logistic regression analysis for the recurrence rate and good functional outcome. Statistical analyses were conducted using IBM SPSS Statistics for Windows version 22.0. All tests were two-tailed and results were considered to be significant at *P*-value <0.05.

## Results

From May 2017 to March 2019, 642 patients with acute ischemic stroke were enrolled in this study (277 in the control group, 365 in the intervention group). Table 1 shows the baseline data of the participants. The baseline characteristics of the participants that are included in analysis were well balanced.

**Table 1:** Baseline characteristics of the study participants.

	**Control group (*N* = 277)**	**Intervention group (*N* = 365)**	**Statistic**	***P***
Demographics				
Age, years ± SD	58.6 ± 10.0	59.8 ± 10.7	−1.483	0.139
Male, *n* (%)	221 (79.8)	290 (79.5)	0.011	0.918
Medical history, *n* (%)				
Ischemic stroke	83 (30.0)	94 (25.8)	1.398	0.237
Diabetes	72 (26.0)	96 (26.3)	0.008	0.930
Hypertension	172 (62.1)	241 (66.0)	1.062	0.303
Dyslipidemia	88 (31.8)	113 (31.0)	0.048	0.826
Coronary artery disease	24 (8.7)	36 (9.9)	0.267	0.605
Myocardial infarction	3 (1.1)	9 (2.5)	1.642	0.200
Atrial fibrillation	5 (1.8)	11 (3.0)	0.947	0.331
Smoking	167 (60.3)	210 (57.5)	0.493	0.483
Drinking	142 (51.3)	195 (53.4)	0.295	0.587
Baseline values ± SD				
SBP (mmHg)	154.0 ± 20.6	153.3 ± 21.0	0.301	0.763
DBP (mmHg)	89.1 ± 12.1	88.0 ± 13.9	0.835	0.404
Blood glucose (mmol/L)	8.11 ± 2.79	8.00 ± 2.87	0.245	0.807
LDL-C (mmol/L)	2.50 ± 0.76	2.62 ± 0.78	−1.860	0.063
NIHSS, median (IQR)	2 (0-5)	2 (0–7)	−1.743	0.081^*^
Length of stay, *d*	10.2 ± 4.2	10.8 ± 4.0	−1.635	0.103

* *Groups were compared by Mann-Whitney U test*.

*SD, standard deviation; SBP, systolic blood pressure; DBP, diastolic blood pressure; LDL-C, low-density lipoprotein cholesterol; NIHSS, National institute of health stroke scale; d, day; IQR, interquartile range*.

At 3 months, both the SBP [147.1 mmHg, 95% confidence interval (CI) 144.8–149.5], DBP (85.2 mmHg, 95% CI 83.6–86.9), and blood glucose level (7.30 mmol/L, 95% CI 6.94–7.66) in the intervention group was lower than in the control group (*P* < 0.05, Table 2). The LDL-C control in the intervention group (2.07 mmol/L, 95% CI 2.01–2.14) was better than in the controls (2.27 mmol/L, 95% CI 2.17–2.37, *P* = 0.001).

**Table 2:** Outcomes at 3 months between control and intervention group.

**Outcome**	**Mean ± SD**		**Statistic**	***P***
	**Control group**	**Intervention group**		
SBP (mmHg)	152.1 ± 19.1	147.1 ± 18.6	2.630	0.009
DBP (mmHg)	89.0 ± 10.7	85.2 ± 11.1	2.555	0.011
Blood glucose (mmol/L)	8.05 ± 2.21	7.30 ± 1.79	2.341	0.021
LDL-C (mmol/L)	2.27 ± 0.80	2.07 ± 0.62	3.382	0.001

*SD, standard deviation; SBP, systolic blood pressure; DBP, diastolic blood pressure; LDL-C, low-density lipoprotein cholesterol*.

Table 3 shows the percentage of mRS score ≤ 2, Barthel index ≥ 60 at 3 months and recurrence rate at 3, 6 months after discharge. There was no significant difference in good functional outcomes at 3 months. Recurrence rate was lower in the intervention group than the control group [3 months, 1.1 vs. 3.6%, odds ratio, 3.672 (95% CI, 0.947–13.897); 6 months, 3.8 vs. 7.9%, odds ratio, 1.976 (95% CI, 0.943–4.142)], but there was no statistical difference.

**Table 3:** Multivariate analysis of recurrence rate, mRS and Barthel index among control and intervention group.

	**Control group (*N* = 277)**	**Intervention group (*N* = 365)**	Odds ratio (95% CI)^*^	***P***
Recurrence rate				
3 months (%)	10 (3.6)	4 (1.1)	3.627 (0.947–13.897)	0.060
6 months (%)	22 (7.9)	14 (3.8)	1.976 (0.943–4.142)	0.071
mRS ≤ 2 (%)	223 (80.5)	287 (78.6)	1.243 (0.724–1.805)	0.565
Barthel ≥ 60 (%)	272 (98.2)	361 (98.9)	2.238 (0.552–9.079)	0.259

*CI, confidence interval; mRS, modified Rankin Scale*.

* *Adjusted for patient characteristics, including age, hyperlipidemia, and National Institutes of Health Stroke Scale score at admission*.

The overall persistence for secondary prevention medications at 3 months after discharge in the intervention group (303/365, 83.01%) was significantly higher than in the control group (201/277, 72.56%, *P* = 0.001) (Table 4). The proportions of patients in the two groups receiving secondary preventive therapies was similar at discharge. At 3 months, 96.05 and 92.25% of participants taking antiplatelet medications in the intervention and control groups, respectively (*P* < 0.05). The corresponding proportion were 77.66 vs. 61.43% (*P* < 0.05) for hypoglycemic agents. Compared to the control group, the persistence of patients taking statins significantly higher in the intervention group: 95.20 vs. 88.72% (*P* < 0.005). There were no differences in the use of antihypertensive agents.

**Table 4:** Three-months persistence of stroke secondary prevention medications.

	**Control group (*N* = 277)**	**Intervention group (*N* = 365)**	**Statistic**	***P***
Overall persistence, *n* (%)	201 (72.6)	303 (83.0)	10.192	0.001
**Antiplatelet agents**				
At discharge	271	354		
3 months, *n* (%)	250 (92.3)	340 (96.1)	4.180	0.041
**Antihypertensive agents**				
At discharge	124	143		
3 months, *n* (%)	109 (87.9)	123 (86.0)	0.208	0.648
**Statins**				
At discharge	257	354		
3 months, *n* (%)	228 (88.7)	337 (95.2)	8.986	0.003
**Hypoglycemic agents**				
At discharge	70	94		
3 months, *n* (%)	43 (61.4)	73 (77.7)	5.106	0.024

*At discharge, the number of patients taking the drug according to the doctor's advice at discharge; 3 months, the number and percentage of patients still using the drug at 3 months of follow-up after discharge*.

## Discussion

In this study, we found that under the intervention of the stroke health manager, the blood pressure, blood lipid and glucose control in the intervention group was better, and the compliance of medicine use was improved compared to the control group.

The control of risk factors was closely related to the recurrence of stroke. The research of PROFESS (Prevention Regimen for Effectively Avoiding Second Strokes) and CNSR (China National Stroke Registry) showed that patients with hypertension had a higher risk of stroke recurrence than non-hypertensive patients (2, 13). In intervention experiments for other types of diseases, such as cardiovascular disease and type 2 diabetes mellitus (14, 15), it was reported that secondary preventive interventions led by a nurse could lead patients to reach target BP, increased physical activity, and significantly reduced the long-term disease risk compared with conventional care (3). Our results revealed that although the blood pressure of patients decreased slightly after intervention by the stroke health manager, the overall control effect was unsatisfactory, and the mean SBP was still higher than 140 mmHg. In addition, there was no difference in patients' compliance with antihypertensive agents between the control and intervention groups—in agreement with an updated review that included 16 new studies (16)—probably because patients with hypertension do not feel uncomfortable and therefore conclude that they are “healthy,” or because they see the condition as temporary (17). Thus, long-term efforts of the stroke health manager are needed.

Patients in the intervention group showed much better LDL-C control than those in the control group, and more insisted on statins. The compliance rate was 95.20% after intervention of this survey—higher than domestic (37.85%) (6) and in South Korea (65.6%) (18). The main reason may be that the address any concerns about medication through WeChat, constantly urged patients to adhere to medication, which improved adherence.

After adjusting the characteristics of the patients, the intervention group showed a decrease in the recurrence rate at 3, 6 months after discharge. The overall incidence of recurrence was similar to the randomized controlled study of Wang et al. (4). The reason why there was no statistical difference between the intervention and control groups may be related to the low incidence of recurrent stroke. Besides, the study samples were small.

However, there were some limitations in our study. First, anticoagulation therapy in patients with atrial fibrillation is very important content in secondary prevention of ischemic stroke, however, the stroke health manager in our center are not doing enough in this area. To make up for this deficiency, stroke health managers are paying more attention to educating patients about atrial fibrillation and anticoagulation recently, and encourage patients to complete 24 h holter monitor as soon as possible after admission to determine the presence or absence of atrial fibrillation. In addition, we have recently established an atrial fibrillation and anticoagulation clinic in conjunction with the cardiovascular department to guide the treatment of patients with atrial fibrillation; the stroke health manager is responsible for the in-hospital and post-discharge management of these patients. Second, the data came from a single center and used historical controls for the comparison cohort. We selected patients who went to the follow-up clinic for face-to-face follow-up, and their compliance might be better. Some of the patients that had received the intervention of the health manager were not included in the study due to the lack of follow-up information at 3 months after discharge. Third, the follow-up time was 3 months; therefore, we are unable to speak to the long-term drug compliance of patients. Fourth, because medication persistence was self-reported, our data may have been biased by the patients' subjective responses.

## Conclusion

Under the intervention of the stroke health manager, the 3-months BP, LDL-C, glucose levels and persistence of secondary prevention medications improved. Stroke health managers may have the function to accelerate the transformation from disease treatment to health management, help stroke patients improve disease awareness, achieve scientific and standardized health management.

## Data Availability Statement

The datasets generated for this study are available on request to the corresponding author.

## Ethics Statement

The studies involving human participants were reviewed and approved by Ethics Committee of the First Hospital of Jilin University. The patients/participants provided their written informed consent to participate in this study.

## Author Contributions

YY and HS conceived and oversaw the study. XY, YS, HJ, XS, and ZL performed data collection. ZL and Z-NG performed data analysis. ZL and XY wrote manuscript.

### Conflict of Interest

The authors declare that the research was conducted in the absence of any commercial or financial relationships that could be construed as a potential conflict of interest.
